# Peste des Petits Ruminants Virus, Tunisia, 2012–2013

**DOI:** 10.3201/eid2012.141116

**Published:** 2014-12

**Authors:** Soufien Sghaier, Gian Mario Cosseddu, Sonia Ben Hassen, Salah Hammami, Héni Haj Ammar, Antonio Petrini, Federica Monaco

**Affiliations:** Istituto Zooprofilattico Sperimentale dell’Abruzzo e del Molise G. Caporale, Teramo, Italy (G.M. Cosseddu, A. Petrini, F. Monaco);; Institut de la Recherche Vétérinaire de Tunisie, Tunis, Tunisia (S. Sghaier, S. Ben Hassen);; Ecole Nationale de Médecine Vétérinaire de Sidi Thabet, Sidi Thabet, Tunisia (S. Hammami);; Ministère de l’Agriculture, Tunis (H. Haj Ammar)

**Keywords:** peste des petits ruminants virus, viruses, molecular characterization, Tunisia

**To the Editor:** Peste des petits ruminants (PPR) is a viral disease of sheep and goats caused by peste des petits ruminants virus (PPRV), a negative-sense, single-stranded RNA virus of the genus *Morbillivirus*. Illness and death can be high (>90%) when PPR occurs in populations of immunologically naive sheep and goats (*1*). Mortality rates are ≈10%–40% in disease-endemic areas (*2*). Because of its economic effects and ability to spread rapidly, PPR has been included among reportable diseases by the World Organization for Animal Health.

In the past 20 years, PPR has shown rapid spread throughout large areas of Africa and Asia (*2*). A unique serotype of PPRV circulates and is classified into 4 genetically distinct lineages (*3*). The geographic distribution of lineages I and II is restricted mainly to western and central Africa and that of lineage III to eastern Africa. Lineage IV is more widely distributed throughout eastern Africa (*4**,**5*), the Near and Middle East, and large areas of Asia (*3*).

In 2008, PPR occurred in Morocco, and 257 cases were reported in goats and sheep over a 6-month period (*3*). In 2011, PPR was officially reported in Algeria (*3*). Genetic analysis showed that PPRV strains isolated in Morocco and Algeria belonged to lineage IV (*4**–**6*). Although PPR has been reported in Tunisia since 2011 (*7*), no data are available on the molecular characterization of PPRV circulating in this country.

During September 2012–January 2013, clinical signs compatible with PPR in ovine and caprine flocks were reported to the Tunisian veterinary service. Ocular, nasal, oral, and rectal swab specimens were obtained from animals showing clinical signs of this disease. Swab specimens were sent to the Institute de la Recherche Vétérinaire de Tunisie in Tunis for laboratory confirmation. Total RNA from swab samples was extracted by using the NucleoSpin RNA Virus Kit (Macherey-Nagel, Düren, Germany) according to the manufacturer’s instructions. The presence of the PPR viral RNA was determined in samples by using a specific reverse transcription PCR reported by Polci et al. (*8*). Laboratory tests confirmed circulation of PPRV in farms near Kairouan and Sidi Bouzid.

Aliquots of RNA samples were shipped to the Istituto Zooprofilattico Sperimentale dell’Abruzzo e del Molise in Teramo, Italy, for genetic characterization. RNA was amplified by using the reverse transcription PCR reported by Couacy-Hymann et al. (*9*). Amplicons from virus- positive samples were purified by using the QIAquick PCR Purification Kit (QIAGEN, Valencia, CA, USA) and used for direct sequencing. Sequencing reactions were performed by using the Big Dye Terminator Kit (Applied Biosystems, Foster City, CA, USA), and nucleotide sequences were determined by using the ABI PRISM 3100 DNA sequencer (Applied Biosystems). Amplification and sequencing were repeated twice to avoid introduction of artificial substitutions. Raw sequence data were assembled by using Contig Express (Vector NTI suite 9.1; Invitrogen, Carlsbad, CA, USA), and a 351-nt fragment of the nucleoprotein coding sequence was obtained after deletion of primer sequences.

Two sequences were obtained, 1 from the Kairouan outbreak and 1 from the Sidi Bouzid outbreak. The 2 sequences generated in this study were submitted to GenBank (accession nos. KM068121 and KM068122). Sequences showed nearly complete identity at nucleotide and amino acid levels; there was 1 nucleotide substitution. The BLAST (http://www.ncbi.nlm.nih.gov) was used to detect homologous regions in sequence databases. Sequences were aligned by using ClustalW (http://www.genome.jp/tools/clustalw/) and MEGA version 6 (*10*). Phylogenetic analysis was performed with a 255-nt sequence of the PPRV nucleoprotein gene by using the neighbor-joining method with bootstrap support (1,000 replicates) in MEGA version 6 (*10*) and reference strains representing the 4 lineages of PPRV that have been isolated in different years or countries (Figure).

**Figure:**
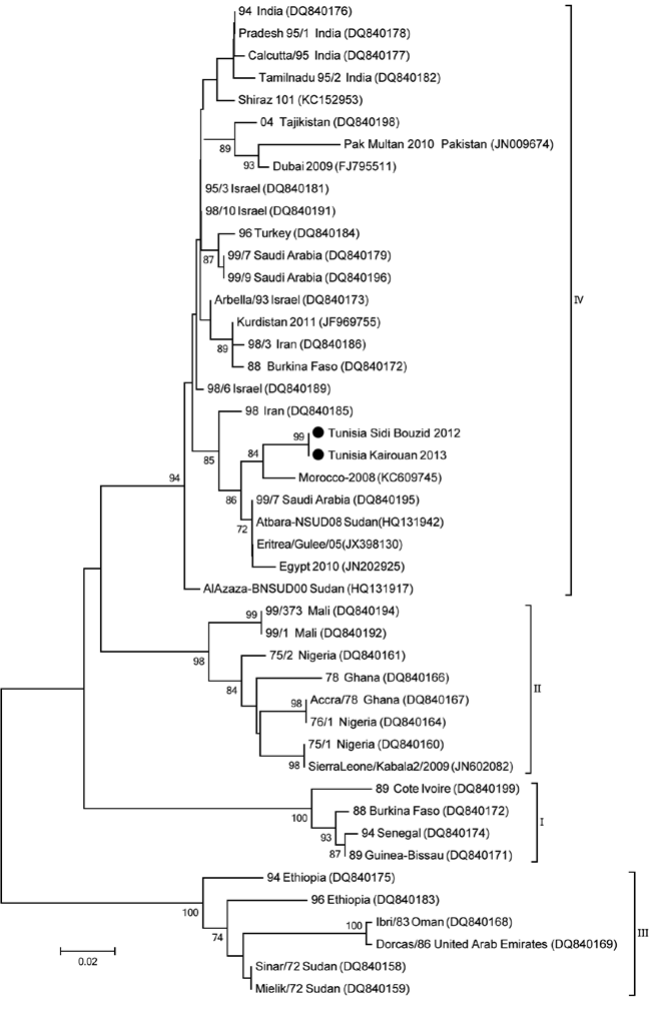
Phylogenetic tree showing genetic relationships among peste des petits ruminants virus (PPRV) isolates. The tree was constructed on the basis of a 255-nt fragment of nucleoprotein gene of PPRV. Sequences obtained in this study are indicated by black circles. Lineages are indicated on the right. Viruses were identified by using the nomenclature of PPRV isolates (GenBank accession numbers are indicated in parentheses). Analysis was performed by using MEGA 6 software (*10*) and neighbor-joining (maximum composite likelihood) methods. Bootstrap support values >70 are shown (1,000 replicates). Scale bar indicates nucleotide substitutions per site.

Our results indicate that lineage IV of PPRV is present in Tunisia. PPRV isolates from the outbreaks in Sidi Bouzid in 2012 and Kairouan in 2013 are closely related to viruses responsible for PPR outbreaks in Morocco in 2008 (*4*) and Algeria in 2010 (*6*). These isolates are closely related to strains from Saudi Arabia, which were detected in Eritrea in 2005 (*5*) and in Sudan in 2008 (*4*). These data suggest that a unique PPRV strain is circulating across this area of the Maghreb. PPRV circulation is maintained probably by the abundant trade in ruminants between Tunisia and neighboring countries. This information highlights the need for a regional approach to control PPR in northern Africa.
